# Characterization of Indomethacin Release from Polyethylene Glycol Tablet Fabricated With Mold Technique

**DOI:** 10.4103/0250-474X.62255

**Published:** 2010

**Authors:** A. Mesnukul, K. Yodkhum, J. Mahadlek, T. Phaechamud

**Affiliations:** Department of Pharmaceutical Technology, Faculty of Pharmacy, Silpakorn University, Nakhon Pathom 73000, Thailand

**Keywords:** Indomethacin, release, mold tablet, polyethylene glycol, xanthan gum

## Abstract

The purpose of this study was to use polyethylene glycol as a carrier to improve the solubility of an aqueous insoluble drug by melting and molding method. The release of dissolved drug was designed to be subsequently sustained with an addition of xanthan gum. The release of indomethacin from the developed system into phosphate buffer pH 6.2 was conducted using the dissolution apparatus. This carrier system could effectively enhance the solubility of indomethacin and an addition of xanthan gum could sustain the drug release. Eudragit L100 film coating could protect the carrier not to be disturbed with HCl buffer pH 1.2 and could dissolve in phosphate buffer pH 6.2, therefore, the drug release from coated tablet was initially very low but subsequently gradually released and prolonged in phosphate buffer pH 6.2. Differential scanning calorimetry study indicated the amorphous state of drug in polyethylene glycol carrier. Scanning electron microscopy photomicrograph indicated the drug diffusion outward through the porous network of matrix tablets into the dissolution fluid and curve fitting signified that the drug release kinetic was Fickian diffusion.

Drug solubility is one of the factors affecting drug bioavailability; therefore many efforts were made to increase solubility of poorly water-soluble drugs. Solid dispersion (SD) of medicaments in an inert carrier in the solid state could be prepared by melting, dissolution in a solvent, or melting solvent method to increase the apparent solubility or enhance the bioavailability of poorly water-soluble compounds[1,2]. In particular, solid polyethylene glycols (PEGs) have been employed as carriers for increasing the dissolution rate of several poorly water-soluble drugs[3–7]. PEG has potentially been utilized as a matrix component prepared with fusion and mold method[8]. All grades of PEG are miscible in all proportions with other PEGs. Therefore, the PEG 4000 combined with PEG 400 was used in this investigation as a carrier for indomethacin which is used as a model poorly water-soluble drug.

Both the enhancement of dissolution and the prolonged drug release of indomethacin could be achieved by addition of hydroxypropylmethylcellulose into PEG matrix system[8]. From that previous report the least square curve fitting indicated that the drug liberated from this matrix system conformed to an anomalous transport. For the present study we are interesting to use xanthan gum as the release retardant of PEG matrix. Xanthan gum has been described as an effective excipient for sustained release dosage forms since it can retard the drug release and sometimes provides the time-dependent release kinetics[9–11].

In this study, indomethacin solid dispersion tablets were prepared with melting and mold method using 7:3 PEG4000:PEG 400 as inert carrier and xanthan gum as hydrophilic polymer to obtain the model formulation having functions of both the drug solubility enhancement and the extended release of the dissolved drug molecules. Effect of xanthan gum, pH of dissolution medium and rotational speed of basket and tablet coating with Eudragit L 100 on the physical properties and *in vitro* drug-release behavior of the systems were investigated.

## MATERIALS AND METHODS

Indomethacin (Batch No. 050814, China National Chemical Imp. Exp., China) was used as received. Polyethylene glycol 4000 (lot no. 504907) and polyethylene glycol 400 (lot no. PO76049) were purchased from P. C. Drug Center Co., Ltd., Thailand. Di-sodium hydrogen orthophosphate (lot no. 405300, Ajax Finechem, Australia), hydrochloric acid (lot no. E23W60, J. T. Baker, USA), potassium dihydrogen orthophosphate (lot no. E23W60, Ajax Finechem, Australia), sodium chloride (lot no. AF 407256, Ajax Finechem, Australia), sodium hydroxide (lot no. AF 310204, Ajax Finechem, Australia) were used to prepare the dissolution fluids. Xanthan gum (Xantural 75^®^, lot no. 01-100, CP Kelco US., Inc. USA.), Eudragit L 100 (lot no. 1200403005, Rohm GmbH Chemische Fabrick, Germany), ethyl alcohol absolute (lot no. V5C933235C, Rohm GmbH Chemische Fabrick, Germany) and triethyl citrate (lot no. 0000078425, Vertellus, USA) were used as received.

### Tablet preparation:

Tablets containing 75-mg indomethacin, PEG4000:PEG400 (7:3) and different amount of xanthan gum were prepared using the melting and mold technique with the stainless steel mold with diameter and height of 12 mm and 6.8 mm, respectively, as described in the previous report[8].

### Hardness, thickness, diameter and drug release from tablet:

The hardness of the tablets was determined using a hardness tester (Pharmatest, USA, n=10). The tablet thickness and diameter were measured using a thickness tester (Teclock, Japan, n=10). A test of drug release was undertaken in 900 ml phosphate buffer pH 6.2 using a dissolution apparatus (Erweka DT 70, Germany) with the basket method at 100 rpm. For drug release test in acid environment, 0.1 N HCl (pH 1.2) was used as dissolution fluid. In the case of the dissolution test with pH change, the drug released in 900 ml 0.1 N HCl was conducted for 1.5 h. Then the pH was increased to 6.2 and continued up to 8 h (1.5+6.5). The contents of indomethacin in sample were determined by measuring the absorbance at 323 nm (n=3) in a UV/Vis spectrophotometer (Perkin-Elmer, Germany). Drug release from these tablets was compared to capsule containing 75-mg indomethacin powder. Least square fitting of the experimental dissolution data to the mathematical equations (power law, first-order, Higuchi's and zero-order) was carried out with the same method as previously described[8,12].

### Content uniformity:

To determine the content of indomethacin in the tablet, an accurate weight sample (1 g) was ground and transferred to a 250 ml volumetric flask containing 1:1 methanol and phosphate buffer pH 7.5. The flask was sonicated for 30 min and then stored at ambient temperature. Two millilitres of the stock solution was pipetted and adjusted to volume in a 25 ml volumetric flask, filtered and analyzed spectrophotometrically at 323 nm (n=10).

### Water sorption and erosion studies:

Investigation of water uptake and erosion of the prepared tablets was performed as follows. The weight of tablet (W_0_) was determined before the test, and then the tablet was put into the basket and immersed in 900 ml phosphate buffer pH 6.2. The basket was rotated at 100 rpm in the dissolution apparatus. At selected time intervals, tablets were withdrawn, blotted to remove excess water, and the wet weight (W_1_) obtained, then they were placed in a hot air oven at 50° until the tablet weight did not change and the dry weight (W_2_) was obtained. The swelling and erosion characteristics of the tablets were expressed in terms of %water uptake and %erosion as reported elsewhere[13]. Water uptake (%) = ((W1-W2)/W2)×100, Erosion (%) = ((W0-W2)/W0)×100

### Morphological studies:

The morphological change, which occurred in the structure of the matrix when it came into contact with the dissolution fluids, was studied with a visual observation. Tablet was fixed between two transparent acrylic plates (6×6 cm^2^). These two plates were linked together with stainless steel screws. The fixed tablet was placed in the dissolution vessel containing 900 ml phosphate buffer pH 6.2 and operated using the paddle method at 50 rpm. The distance between the paddle and the vessel bottom was adjusted to 2/3 of the compendial height (5.5 cm). To analyze the morphological behavior of the systems during the release process, tablets were withdrawn from the dissolution vessels at different time intervals and their photographs were recorded using a digital camera (Samsung Digimax i5, Korea). The surface topography of the prepared matrix tablet was also determined using scanning electron microscope (SEM, Maxim 200 Camscan, Cambridge, England). The sample was lyophilized prior to test with SEM.

### Texture analysis:

Swelling behavior of the tablets was investigated through textural analysis as described in the previous report[8,14]. Briefly, tablets were placed in the dissolution vessels under condition identical to that described above for dissolution testing. Textural analysis was performed using a TA.XT2i texture analyzer (Charpa Techcenter, Godalming, Stable micro Systems Ltd., UK) equipped with a 5 kg load cell and Texture Expert software. The force displacement–time profiles associated with the penetration of a 3 mm round-tipped steel probe into the swollen matrices were monitored at a data acquisition rate of 200 points per second. Probe approached the sample at a pretest speed of 1.0 mm/s. Once a trigger force of 0.005 N was detected (at contact of the probe with tablet) the probe was advanced into the sample at a test speed of 0.5 mm/s until the maximum force of 40 N was reached (n=3).

### Differential scanning calorimetry (DSC):

The DSC thermograms of drug, PEG 4000, PEG 400, xanthan, tablet containing PEG 4000:PEG 400 and tablet containing 25% xanthan gum were obtained using differential scanning calorimetry (DSC, Pyris Sapphire DSC, Standard 115V, Perkin Elmer instruments, Japan) with the method and condition described previously[8].

### Tablet coating:

Eudragit L100 solution (5% w/w) was prepared by dissolving the polymer in absolute ethanol. Triethyl citrate at 30% w/w of dry polymer was added in polymer solution as plasticizer. Tablets were coated by dipping method. The core tablet was completely dipped into the polymeric solution and then dried stepwise with a hair dryer. The % increase in weight of tablet was 4% w/w after coating. Drug release from the obtained coated tablets was studied and fitted with different release equations. Surface of film coated tablet was also determined using scanning electron microscope (SEM).

### Statistical analysis:

Data were analyzed using SPSS software. Analysis of variance (ANOVA) and Duncan's multiple range method were used to compare any significant differences between test parameters and samples. Values were expressed as mean±standard deviations. Differences were considered significant at *P*<0.05.

## RESULTS AND DISCUSSION

Hardness of tablet containing different amounts of xanthan gum increased as the polymer amount was increased (Table 1). Most remarkable properties of xanthan gum were its capability to produce a large increase in viscosity with a very small quantity of gum[15,16]. Xanthan solutions are highly viscous even at low polymer concentrations[17]. This property is useful in many industrial applications, especially in the food industry where xanthan is used as a thickener and to stabilize suspensions and emulsions. Xanthan gum should act as binder in this system and promote the hardness of the prepared tablets. Drug content of the matrix tablets containing different amounts of xanthan gum met the requirement of the test which the labeled amount was in the range of 93.08±1.24% and 94.61±1.51% of indomethacin. This indicated the good homogeneity during blending of mixtures before pouring into the mold.

**TABLE 1:** PHYSICAL PROPERTIES OF 75-MG INDOMETHACIN TABLETS

Amount of xanthan gum (%)	Weight±SD (g)	Thickness±SD (mm)	Diameter±SD (mm)	Hardness±SD (Newton; N)
0	0.8551±0.0146	6.73±0.21	11.91±0.03	14.62±1.75
5	0.8709±0.0089	6.82±0.22	12.03±0.02	15.22±1.65
10	0.8685±0.0098	6.73±0.22	12.02±0.03	16.68±1.73
15	0.8739±0.0098	6.65±0.21	12.00±0.02	17.64±1.78
20	0.8810±0.0228	6.82±0.19	12.03±0.02	18.43±1.78
25	0.8762±0.0151	6.72±0.19	12.02±0.02	18.95±1.83

Physical properties of 75-mg indomethacin tablet containing 7:3 PEG4000:PEG400 and different amount of xanthan gum

**Fig. 1:**
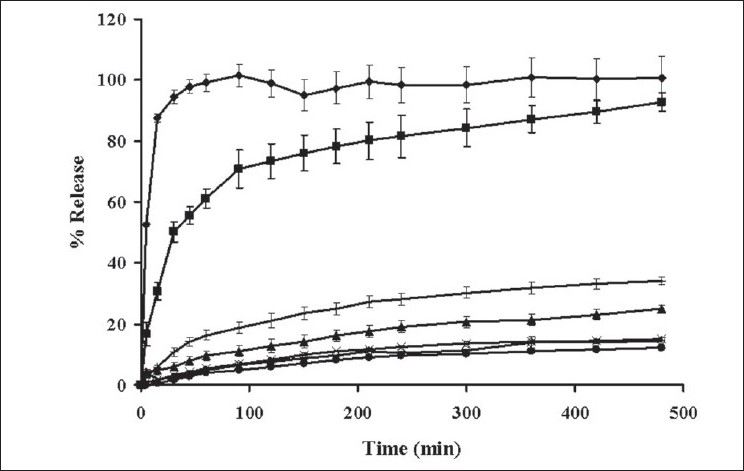
Drug release profiles of indomethacin from capsule and tablets Drug release profiles of indomethacin from (–+–) capsule and tablets containing (–♦–) 0% xanthan gum; (–■–) 5% xanthan gum; (–▲–) 10% xanthan gum; (–×–) 15% xanthan gum; (–○–) 20% xanthan gum; (–●–) 25% xanthan gum in phosphate buffer pH 6.2 (n=3)

The dissolution of indomethacin from SD tablet containing different amounts xanthan gum is shown in fig. 1. Drug release from tablet without an addition of xanthan gum was faster than that from tablet containing other amounts of xanthan gum because the former tablet contained PEG as carrier to increase the solubility of indomethacin. Drug release form tablet prepared by solid dispersion method was apparently faster than that from capsule containing only 75 mg indomethacin. PEGs are one of the carriers most widely used to prepare solid dispersions due to their low melting point and their ability to provide the hydrophilic environment to enhance the drug solubility[3]. Xanthan gum at higher concentrations, on exposure to dissolution fluids, hydrated and formed a viscous gel layer that slowed down further seeping-in of dissolution fluids towards the core of the matrix tablet. In addition, the synergism attributed to the intermolecular hydrogen-bonding between acid group of indomethacin and OH-group of xanthan gum could occur[18]. High swelling capacity and gel formation of xanthan gum could retard the release of dissolved drug from the matrix. Relationship between drug release rate and polymer concentration was previously reported[19].

**Fig. 2:**
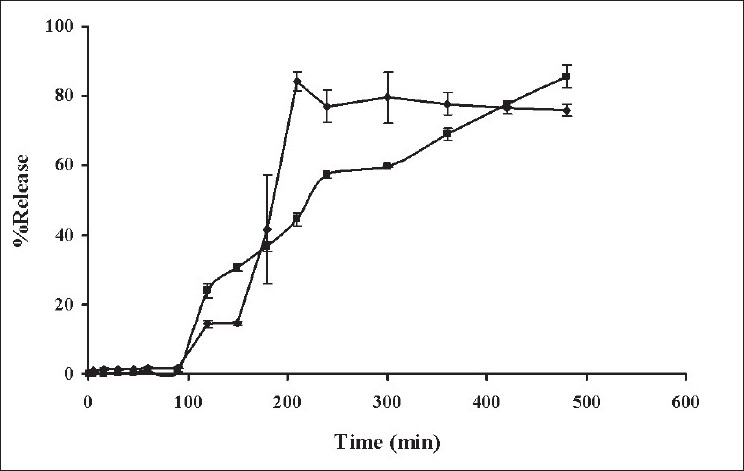
Drug release profiles of indomethacin from tablets Drug release profiles of indomethacin from (–♦–) tablet containing 5% xanthan gum and (–■–) tablet containing 5% xanthan gum coated with Eudragit L100 in pH change system (n=3)

Drug release from tablet coated with Eudragit L 100 was compared to that of tablet without Eudragit L 100 coating in pH change system (fig. 2). The drug release from tablet without Eudragit L 100 coating was very low but abruptly increased as the pH of medium was enhanced to 6.2 and the drug release was constant after 4 h. In contrast, the initial release of tablet coated with Eudragit L 100 was very low but the drug was gradually released and could be prolonged in phosphate pH 6.2. This result indicated that Eudragit L100 film could be used to deliver drug to small intestine and drug was subsequently gradually liberated. Eudragit L 100 is the commercial name of an enteric polymer of methacrylic acid-methylmethacrylate that is designed to provide a protection to tablets in the stomach[20,21]. The tablet was coated with Eudragit L 100 to protect the stomach from being irritated by indomethacin and deliver this drug to small intestine. Furthermore, the film coat would protect the carrier, PEG, not to be disturbed with 0.1 N HCl before the tablet was moved into the intestine. The indomethacin tablet coated with Eudragit L 100 demonstrated the low initial release of drug in 0.1 N HCl of pH change system. Eudragit L 100 is a pH-dependent polymer which dissolves in medium with pH> 6 and is less soluble in medium with pH 1.2[20,21]. The release of drug was gradually increased up to 90% in phosphate buffer pH 6.2 because Eudragit L 100 could dissolve in this environmental pH and the drug release was sustainable with the gelation of xanthan gum.

To analyze the *in vitro* release data, the curve fitting of drug dissolution profiles to various kinetic models was carried out to describe the release kinetics. The large value of coefficient of determination (r^2^) or model selection criteria (MSC) indicated a superiority of the dissolution profile fitting to mathematical equations. The r^2^ and MSC from curve fitting to power law, first order, Higuchi's and zero order equations are shown in Table 2. The description for drug release with first order, zero order or Higuchi's equations have been mentioned previously[22–24]. The r^2^ from curve fitting to power law equation ranged from 0.9808 to 0.9989 and msc was in the range of 1.94 to 6.23 (Table 2). The dissolution data of tablet containing different amounts of xanthan gum and tablet coated with Eudragit L100 were fitted well on Higuchi's model. The exponent *(n)* values for almost formula are shown in Table 3. The tablets containing different amounts of xanthan gum and tablets coated with Eudragit L100 tended to exhibit Fickian transport characteristics as corresponding values of *n* were lower than the standard value for declaring Fickian release behavior, i.e. 0.45[25].

**TABLE 2:** COMPARISON OF DEGREE OF GOODNESS-OF-FIT

Formula	Power law		First order		Higuchi's		Zero order	
				
	r^2^	msc	r^2^	msc	r^2^	msc	r^2^	msc
Capsule	0.9913	4.16	0.9955	2.98	0.9811	3.51	0.9915	4.23
5 % xanthan gum	0.9985	5.95	0.8996	1.90	0.8560	1.54	0.7572	1.02
10 % xanthan gum	0.9944	4.73	0.9519	2.73	0.9896	4.26	0.9391	2.49
15 % xanthan gum	0.9816	3.45	0.8806	1.76	0.9449	2.54	0.8762	1.73
20 % xanthan gum	0.9955	5.46	0.8987	1.85	0.9597	2.81	0.9276	2.23
25 % xanthan gum	0.9913	4.98	0.8935	1.80	0.9562	2.68	0.9166	2.04
5% xanthan gum coated with Eudragit L 100	0.9808	3.20	0.9824	3.54	0.9841	3.62	0.9599	2.72

Comparison of degree of goodness-of-fit from curve fitting of drug dissolution profiles in phosphate buffer pH 6.2 to different release models

**TABLE 3:** ESTIMATE PARAMETERS FROM CURVE FITTING

Formula	k±SD×10^-1^	tl±SD (min)	n±SD
Capsule	0.1232±0.0208	3.12±1.03	0.31±0.05
5 % xanthan gum	0.0098±0.0011	64.39±4.23	0.51±0.02
10 % xanthan gum	0.0187±0.0025	14.64±4.70	0.42±0.02
15 % xanthan gum	0.0225±0.0041	46.32±7.05	0.32±0.03
20 % xanthan gum	0.0238±0.0072	28.21±9.38	0.43±0.05
25 % xanthan gum	0.0162±0.0091	15.94±62.13	0.44±0.11
5% xanthan gum coated with Eudragit L 100	0.0338±0.0210	85.59±22.17	0.44±0.10

Estimate parameters from curve fitting of drug dissolution in pH change media to power law expression

The release kinetics of tablets containing different amounts of xanthan gum and tablet coated with Eudragit L100 adequately followed a square root of time according to the Higuchi's model since Fickian diffusion occurred via the porous network formed when indomethacin dissolved from the inert matrix. After exposure to dissolution medium, the macromolecular chain of xanthan gum swelled at the tablets surface and formed a gel layer around a dry-like core. Drug diffusion occurred at the core-gel interface then through this hydrated gel[26]. Erosion of swollen layer and dissolution of the matrix itself were also observed. Typically, the sustained release formulations, diffusion, swelling and erosion were the three most important rate controlling mechanisms. The drug release from these matrices was mostly by diffusion and was best described by Fickian diffusion.

**Fig. 3:**
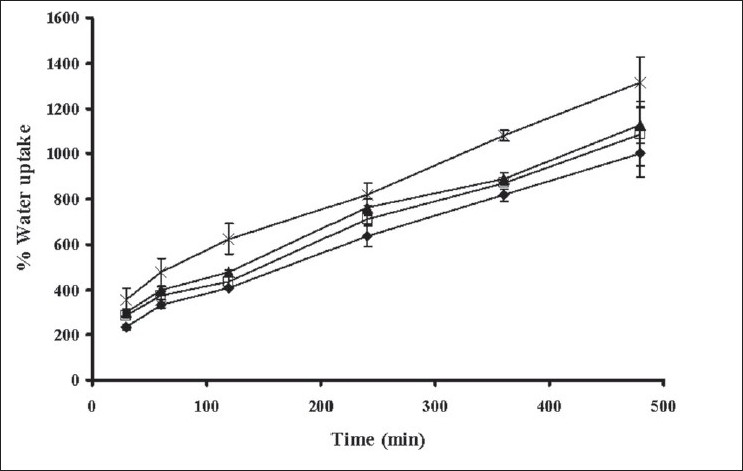
Water uptake of the tablets in phosphate buffer Water uptake of the tablet containing (–♦–) 10% xanthan gum; (–□–) 15% xanthan gum; (–▲–) 20% xanthan gum; (–×–) 25% xanthan gum in phosphate buffer pH 6.2 (n=3)

**Fig. 4:**
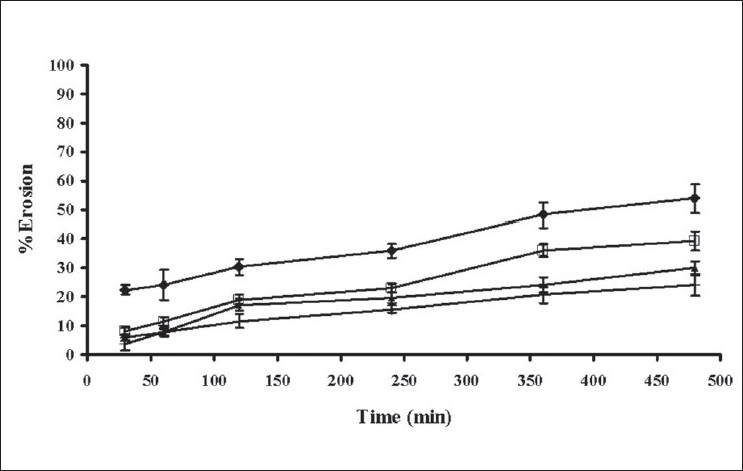
Erosion of tablets in phosphate buffer Erosion of tablets containing (–♦–) 10% xanthan gum; (–□–) 15% xanthan gum; (–▲–) 20% xanthan gum; (–×–) 25% xanthan gum in phosphate buffer pH 6.2 (n=3)

The swelling behavior of tablets containing different amounts of xanthan gum was estimated from water uptake amount of the systems. The percentage water uptake of these matrices increased with time (fig. 3). The percentage water sorption of tablet containing 25% xanthan gum was statistically higher than that of the tablets containing 20, 15 and 10% xanthan gum (significant at *P*<0.05 level), respectively. The erosion of tablet containing 25% xanthan gum was statistically lower than that of tablet containing 20, 15 and 10% xanthan gum, respectively (significant at *P*<0.05 level), (fig. 4). The percent erosion increased with time. The gel layer was formed on the surface of tablet due to swelling of xanthan gum in the presence of water[7]. The maximum swelling ratio was found for the tablet containing the highest amount of xanthan gum. Therefore a high degree of swelling was due to the high water uptake and the small degree of erosion. Percent water sorption of matrices increased progressively with time. The tablet containing the lowest of amount xanthan gum exhibited the lowest swelling ratio and high degree of erosion. Nature of the polymer played an important role in this swelling process and drug release from the matrices[16,27]. Therefore, the drug release was closely related with percent water uptake and erosion.

**Fig. 5:**
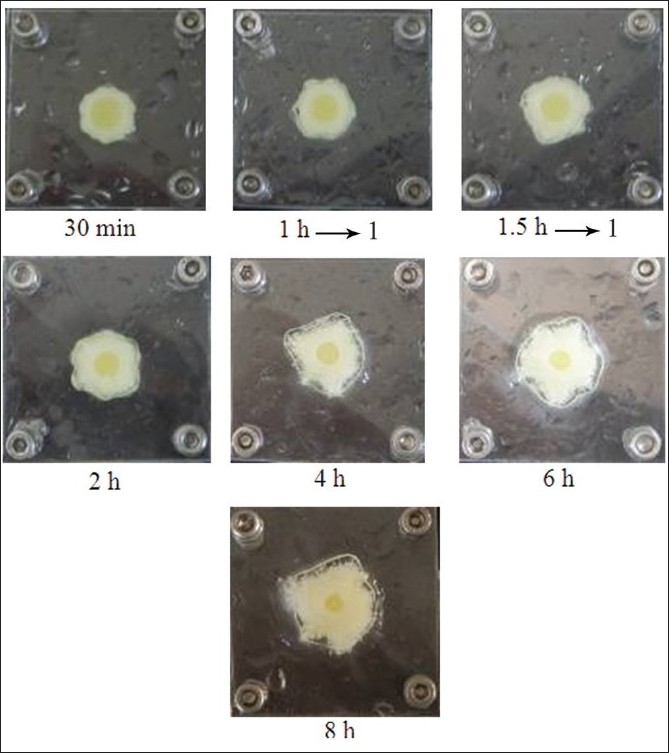
Photographs of indomethacin tablet with time in dissolution test Photographs of 75-mg indomethacin tablet containing 25% xanthan gum after 30 min, 1, 1.5, 2, 4, 6 and 8 of dissolution test in phosphate buffer pH 6.2.

The results from visual observation of tablet containing different amounts of xanthan gum indicated that the matrices appeared to swell and form a viscous gel mass after contacting with the medium. Indomethacin tablets containing 5% and 10% xanthan gum in phosphate buffer pH 6.2 exhibited high hydration at 30 min, 1 h and 1.5 h (data not shown). Similarly, the outer layer of tablet containing 5% and 10% xanthan gum contained the gel layer while the core tablet was completely wet at 2 h. In fact, upon contact with the dissolution fluid, the system hydrated slowly and swelled giving rise to a thick gel layer. The gel thickness increased progressively moving inwards as a function of hydration, the dimensions of the solid core slightly decreased. Photographs of tablets containing 25% xanthan gum are shown in fig. 5. The hydration of tablets containing 15, 20 and 25% xanthan gum was increased with time. Gel thickness increased as a function of hydration whereas the dimension of the solid core gradually decreased.

The photographs indicated a dark yellow at solid core of tablet swelling and soft yellow at gel layer because indomethacin is a pale yellow crystalline powder. Clearly, the intensity of the yellow color indicated the dissolved drug concentration. The color related to the position inside the gel layer. The gradient of color in the gel layer depended on the swelling time. The presence of two concentric layers could be noticed from the system containing high amount of hydrophilic polymers. The external part had a consistency similar to a gel and the inner core was not solid but completely wet. Initial tablet swelling indicated the high hydration of all tablets containing xanthan gum. When a matrix containing a swellable glassy polymer such as xanthan gum contacted with a solvent, a progressive change from the glassy to the rubbery states led to a swelling process[28,29]. The matrix exhibited a rigid and elastic structure after utilizing higher amount of xanthan gum. In contrast, tablet containing low amounts of xanthan gum could not maintain the shape of tablet owing to the high swelling capacity of xanthan during immersion in the dissolution.

**Fig. 6:**
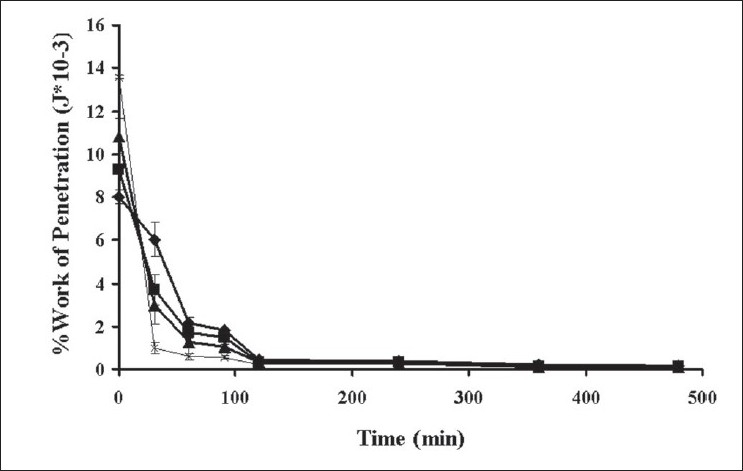
Total work of penetration of tablet in phosphate buffer Total work of penetration of tablet containing (–♦–) 10% xanthan gum; (–■–) 15% xanthan gum; (–▲–) 20% xanthan gum; (–×–) 25% xanthan gum in phosphate buffer pH 6.2 (n=3)

The dynamic structural changes of the gel layer formed during swelling of tablets containing different amounts of xanthan gum were followed by force-displacement measurements (fig. 6). Total work of penetration of tablet containing different amounts of polymer before dissolution test increased with increasing amount of polymers which corresponded with that determined with typical hardness tester as previous reported. The force required for probe to penetrate the swollen tablet decreased with time as the swelling proceeded and gel strength was reduced. The force required at 30 min of all tablets was statistically higher than that at the other periods (significant at *P*<0.05 level). Similar result has been reported for the relation between the this property of a xanthan matrix in the absence or presence of calcium ions on the release of pentoxifylline[14]. Change in work of penetration versus time after exposure to medium was extended as the hydration increased. A sharp decrease in work of penetration from 0 h (dry tablet) to 1 h could be observed which reflected the initially high rate of hydration of tablets. This incidentally coincided with high rate of water uptake and gel formation. Hydrated tablets demonstrated lower values for work of penetration during 2 to 8 h. Total work penetration of tablet containing 25% xanthan gum decreased with time. A sharp decrease in work of penetration reflected the initially high rate of hydration of tablets which incidentally coincided with high rate of water uptake and gel formation. All formulations showed lower values for work of penetration during 2 to 8 h. In fact, the obtained results confirmed that the system containing a gel with a lower strength was greatly susceptible to erosion and chains disentanglement as mentioned previously[30]. Total work of penetration of tablet containing different amounts of xanthan gum was decreased with time. Therefore, the use of texture analysis on the swollen tablet also provided a good approach to understanding drug release kinetics and the mechanism of drug delivery from a swellable matrix system.

**Fig. 7:**
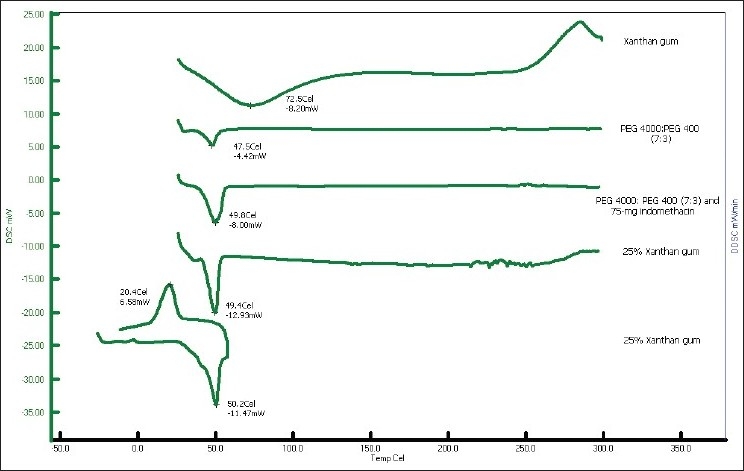
DSC thermograms DSC thermograms of xanthan gum; 7:3 PEG 4000:PEG 400; indomethacin tablet without or containing 25% xanthan gum; reverse run of DSC study for indomethacin tablet containing 25% xanthan gum

Indomethacin, PEG4000 and PEG400 exhibited the endothermic peak at 161.4°, 58.9° and 58.2°, respectively (data not shown). Xanthan gum and 7:3 PEG 4000:PEG400 showed the melting peak at 72.5° and 47.5°, respectively (fig. 7). The thermograms of the solid dispersion showed the characteristic peak of the carrier matrix without drug peak, indicating that the drug was completely dissolved in the molten carrier during DSC measurement. Analogous phenomena have also previously been reported[31–33]. Alteration of some drugs in PEG-based SD system has been reported previously by Zheng *et al*.[29]. The exothermic peak of tablets containing 75 mg indomethacin exhibited at 24.7° after reverse run to −30°. Because the system containing 75 mg indomethacin contained a high amount of carrier, the carrier was recrystallized resulting in the exothermic peak was occurred[34].

**Fig. 8:**
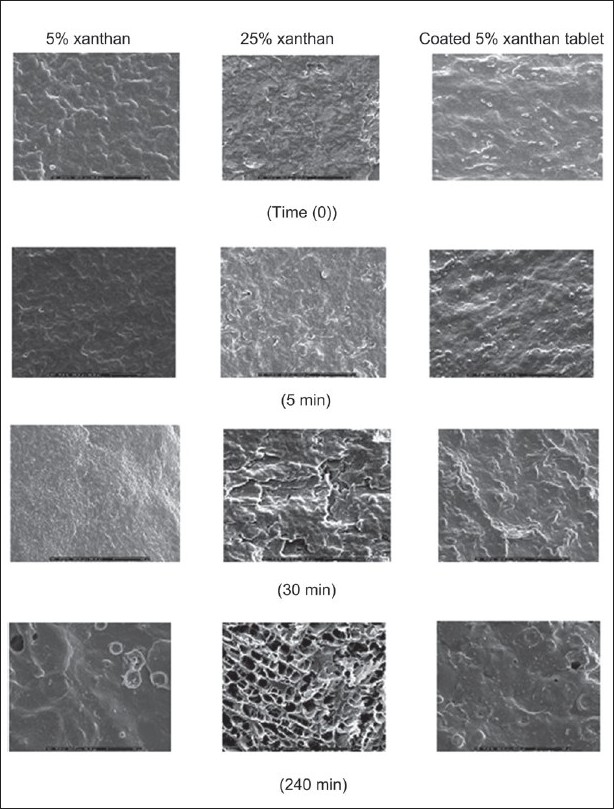
SEM micrographs of indomethacin matrix and coated tablets SEM micrographs of indomethacin matrix and coated tablets after dissolution test in phosphate buffer pH 6.2 at different time interval with magnification of 250

SEM images of indomethacin tablet containing 75 mg indomethacin and 5% xanthan gum before and after dissolution test are presented in fig. 8. Although some particles were observed in the matrix, most drugs should be molecularly dispersed in the PEG4000:PEG400 matrix. The tablet containing 5% xanthan gum exhibited less porosity. SEM images of tablet containing 25% xanthan gum after dissolution test in phosphate buffer pH 6.2 at different time intervals are shown in fig. 8. Porosity of the tablet containing 25% xanthan gum increased with time. The tablet containing the highest amount of xanthan gum showed larger pore formation after drug dissolution. In fact, the increased porosity of the surface promoted the increase of drug diffusion and the opening of the channels. This was reflected in the release studies, wherein the release increased with the increase in level of pore former. However, in this study tablet containing the highest amount of xanthan gum exhibited the lowest drug release from tablet. Other factors like closer contact between the hydrophilic carrier and the drug could also influence and decrease the drug release. The higher amount of PEG promoted the matrix erosion owing to its high solubility, therefore there was no sponge-like structure as exhibited in the case of system containing higher amount of polymer, such as xanthan gum. However, the dissolved drug molecules could diffuse through the pore and the kinetics of drug release should be the diffusion-controlled release[35,36]. Ahuja *et al*.[7] described the solubility enhancement of a poorly soluble model drug, rofecoxib, using solid dispersion approach. Solid state characterization revealed the partial loss of drug crystallinity which could bring about significant change in the drug dissolution rate.

The continuous coated layer was noticed from tablet coated with Eudragit L100 (fig. 7). The distinct coated layer without cracks or pores was visualized before dissolution test and at 5 min after dissolution test. It also showed negligible drug release in the initial 90 min of dissolution. Film from Eudragit L 100-coated tablet was completely dissolved in phosphate buffer pH 6.2. Then the drug was gradually released from tablet and could be prolonged in phosphate pH 6.2.

Successful solubilization of indomethacin was achieved using 7:3 PEG4000:PEG400 as drug carrier for tablet preparation with melting and mold technique. Tablet containing 5% xanthan gum as hydrophilic polymer could prolong the release of indomethacin from tablet. Tablet coated with Eudragit L100 could prolong drug release in pH change system following Higuchi's model.
